# Bulk-Fill Resins versus Conventional Resins: An Umbrella Review

**DOI:** 10.3390/polym15122613

**Published:** 2023-06-08

**Authors:** Gonçalo Silva, Carlos Miguel Marto, Inês Amaro, Ana Coelho, José Sousa, Manuel Marques Ferreira, Inês Francisco, Francisco Vale, Bárbara Oliveiros, Eunice Carrilho, Anabela Baptista Paula

**Affiliations:** 1 Institute of Integrated Clinical Practice, Faculty of Medicine, University of Coimbra, 3000-354 Coimbra, Portugal; goncaloestiveiras@gmail.com (G.S.); cmiguel.marto@uc.pt (C.M.M.); ines.amaros@hotmail.com (I.A.); anasofiacoelho@gmail.com (A.C.); ze-93@hotmail.com (J.S.); eunicecarrilho@gmail.com (E.C.); 2 Laboratory for Evidence-Based Sciences and Precision Dentistry (LACBE–MDP), Faculty of Medicine, University of Coimbra, 3000-354 Coimbra, Portugal; m.mferreira@netcabo.pt (M.M.F.); ines70.francisco@gmail.com (I.F.); fvale@fmed.uc.pt (F.V.); boliveiros@fmed.uc.pt (B.O.); 3 Institute for Clinical and Biomedical Research (iCBR), Area of Environment Genetics and Oncobiology (CIMAGO), Faculty of Medicine, University of Coimbra, 3000-354 Coimbra, Portugal; 4 Institute of Experimental Pathology, Faculty of Medicine, University of Coimbra, 3000-354 Coimbra, Portugal; 5 Clinical Academic Center of Coimbra (CACC), 3000-354 Coimbra, Portugal; 6 Center for Innovative Biomedicine and Biotecnhology (CIBB), 3000-354 Coimbra, Portugal; 7 Institute of Endodontics, Faculty of Medicine, University of Coimbra, 3000-354 Coimbra, Portugal; 8 Institute of Orthodontics, Faculty of Medicine, University of Coimbra, 3000-354 Coimbra, Portugal; 9 Laboratory of Biostatistics and Medical Informatics (LBIM), Faculty of Medicine, University of Coimbra, 3004-531 Coimbra, Portugal

**Keywords:** abrasion, bulk-fill, composite resin, microleakage, modulus of elasticity, polymerisation shrinkage

## Abstract

Currently, composite resins have become the material of choice for the restoration of posterior teeth. Although bulk-fill resins represent a tempting alternative due to their lower complexity and faster use, some dentists are reluctant to use this material. The objective is to compare the performance of bulk-fill resins and conventional resins in direct restorations of posterior teeth based on the literature. The databases that were used to carry out the research were PubMed/MEDLINE, Embase, the Cochrane Library and the WOS. This umbrella literature review complies with PRISMA standards and assesses the quality of studies using the AMSTAR 2 tool. With the application of the criteria of the AMSTAR 2 tool, the reviews were considered low to moderate. The overall meta-analysis, although without statistical significance, favours mostly the use of conventional resin, as it is about five times more likely to obtain a favourable result than bulk-fill resin. Bulk-fill resins result in a simplification of the clinical process of posterior direct restorations, which is an advantage. The performance in terms of several properties of bulk-fill resins and conventional resins showed that they present similar behaviour.

## 1. Introduction

Caries lesions represent the pathology that dentists are most often faced with in oral cavities. These arise due to bacterial activity that promotes demineralisation on the tooth surface, through the production of acidic components that result from the metabolism of nutrients. The recommended treatment for this pathology consists of removing all the carious tissue and subsequently filling the cavity with a suitable restorative material [1,2].

Currently, due to increasing demands and the establishment of higher standards, often strictly related to aesthetic aspects, restorative materials have had to evolve to live up to these expectations. Thus, currently, composite resins have become the material of choice for the restoration of posterior teeth [2].

Although composite resins are a frequently used, promising material, with many advantages compared with their precursor (dental amalgam), they still present some problems [2]. The biggest fear that arises when using this type of material is mainly related to polymerisation shrinkage and the successive mechanical stress that is associated with direct composite resin restorations. Microleakage, loss of adhesion of the restoration to the tooth structure, and, more commonly, secondary caries is some of the examples of the consequences that can arise from the failure of this type of material [3].

Polymerisation shrinkage is the main disadvantage of composite resin restorations. To combat the weaknesses associated with this material, some techniques have been proposed to reduce the stress associated with the material shrinkage and ensure better results. These techniques include the adjustment and modelling of the light intensity in polymerisation, the use of indirect composite resin restorations (whose polymerisation takes place outside the cavity, with only the contraction of the resinous cementing material), the application of a liner of flowable composite resins (for adjustment to the irregularities of the cavity floor), and the use of the incremental technique, which is quite common in direct composite resin restorations [4,5,6]. This last technique is widely recommended, since when smaller amounts (around 2 mm) are placed, successively, oblique and with altered geometry, it is expected that there will be a decrease in the stress related to the C-factor (ratio between the areas of adhered surfaces and the areas of free surfaces). In turn, this consequent decrease will lead to the possibility of decreasing the shrinkage stress on the material [6,7].

The modification of the composite resin insertion was one of the ways found to reduce the effects of polymerisation shrinkage. However, over time, changes in the composition of composite resins were used with the same objective, either in changing the percentage of filler versus resin or in the introduction of other resin monomers, namely ormocer and silorane [8,9,10,11]. 

Bulk-fill resins have emerged with the objective of simplifying the insertion technique with the placement of layers of greater thickness, keeping the polymerisation shrinkage low. They simplify the restorative procedure in posterior teeth, since they allow the use of a single composite resin increment of 4–5 mm, resulting in a less time-consuming procedure than the conventional method [12,13]. This is possible since these composite resins have several specificities that make them ideal for the treatment of posterior teeth. They present greater translucency and, consequently, better light dissipation in the composite resin, with photo initiators allowing a greater polymerisation depth and polymerisation modulators allowing for less polymerisation shrinkage [14].

Bulk-fill resins can be categorised into two groups, base with low viscosity and full-body with high viscosity, depending on the purpose for which they are used, namely the restoration type and its mechanical requirements [6,15].

The first group, having a low viscosity, is easy to sculpt and can be sonically activated to become more fluid and more easily adaptable to the cavity walls [6].

Normally, the application of flowable bulk-fill resins can be carried out using a syringe, since they are characterised by their high fluidity. Thus, the application is simpler, allowing use of the composite resin in cavities that are more difficult to access. However, this type of composite resin is often associated with low strength, and it is necessary to cover it using conventional composite resins, thus hiding the more transparent aspect of the restoration given by bulk-fill composite resins [14,15,16,17].

However, despite bulk-fill resins representing a tempting alternative, due to their lower complexity and faster use when compared with conventional resins, they are still not widely used by clinicians [1].

In the current literature, several studies address the different mechanical properties of bulk-fill composite resins and compare them with conventional resins. However, the results are inconsistent, and it becomes impossible to say with certainty that these composite resins are associated with greater clinical efficacy when compared with conventional resins. Therefore, it is extremely important to review the evidence available in the literature to allow reliable conclusions to be drawn [13,18,19].

The Academy of Dental Materials designates some characteristics as the most important for material performance evaluation, which are discoloration, modulus of elasticity, fracture resistance, fatigue, occlusion resistance, and abrasion and friction wear measurements [20]. The objective of this study is to compare the performance of bulk-fill resins and conventional resins in direct restorations of posterior teeth, evaluating some characteristics directly or indirectly, namely marginal coloration, marginal adaptation, secondary caries, restoration integrity and clinical performance [20]. As there are already several systematic reviews on this topic in the literature, the objective is to analyse the results of all these reviews, hence the option for an umbrella review. In addition to the qualitative analysis of systematic reviews, the objective is also a statistical analysis of the review-extracted data in a meta-analysis.

## 2. Materials and Methods

### 2.1. Protocol Registration

This review was registered in the international prospective register of systematic reviews (PROSPERO) under number 339,190 and conducted according to the COCHRANE guidelines.

### 2.2. Review Question

This umbrella review of the literature was carried out based on the PRISMA guidelines for systematic reviews [21]. The purpose of this review was to evaluate the performance of bulk-fill resins, evaluating several parameters, such marginal coloration, marginal adaptation, secondary caries, restoration integrity and clinical performance. For this purpose, the following PICO question was proposed:

“Are restorations of posterior teeth with bulk-fill resins expected to have superior performance to restorations of posterior teeth with conventional resins?” (Table 1).

### 2.3. Search Strategy

The search strategy was performed independently by two investigators (GS and AP). The databases consulted to carry out the research were PubMed/MEDLINE, Excerpta Medica Database (Embase), the Cochrane Library, the Web of Science and Epistemonikos (Table 2). Systematic review, meta-analysis and language filters (Portuguese and English) were applied. A search was also carried out in the grey literature on the websites Proquest (https://www.proquest.com, accessed on 2 March 2023) and OpenGrey Europe (https://opengrey.eu, accessed on 2 March 2023).

### 2.4. Eligibility Criteria

Inclusion and exclusion criteria were established.

Inclusion criteria were all systematic reviews of randomised clinical studies, non-randomised clinical studies, case-control studies and in vitro studies that compared bulk-fill resins and conventional composite resins and that presented meta-analyses.

Regarding the exclusion criteria, all systematic reviews that did not present a meta-analysis and that did not present a comparison between bulk-fill resins and conventional resins were excluded.

In the first phase, the articles were selected based on the titles and abstracts, according to the eligibility criteria, by the two independent reviewers mentioned above. Subsequently, the full texts were revised for possible inclusion. The final number of articles included was compared between the two authors. In cases of disagreement, a third evaluator (EC) was included in the eligibility process, always obtaining a consensus.

### 2.5. Data Extraction and Meta-Analysis

The data extracted from each article for an initial qualitative analysis were author and year, type of review, number of studies and their design, results of ROB and ROB tool, sample size, evaluated properties and main results.

Studies that reported the number of events in each group for the discoloration or marginal staining, marginal adaptation, secondary caries and restoration integrity outcomes were analysed. For each of these outcomes, two studies were included, one reporting the relative risk and the other reporting the odds ratio. As these measures depend on the study design, and to assess an overall summary measure, the odds ratio was calculated and used for both studies. For the clinical performance outcome, there were three studies, but only one of them evaluated the number of events in each group and thus only this study was considered for the meta-analysis.

Summary measures were obtained using a random effects model despite few heterogeneities between studies detected due to a small sample size in terms of the number of studies involved in computation of summary measures. The Mantel–Haenszel or the inverse variance methods were applied for achieving the odds ratio or mean differences summary measures, respectively, and their 95% confidence intervals. Heterogeneity between studies was evaluated through the Higgins and Thompson I^2^ statistic. Analysis was performed using Review Manager, version 5.4.1, and overall comparisons were performed at a 5% significance level.

### 2.6. Quality and Risk of Bias Assessment

To assess the quality of the studies selected to be included in the study, the AMSTAR2 tool was used. Two investigators (A.B.P. and I.F.) carried out the independent assessment and, in cases of disagreement, a third investigator was included (C.M.M.).

AMSTAR 2 presents 16 items that aim to assess the methodology used in the different studies and the risk of bias (ROB). The studies were scored as high, moderate, low, and critically low quality. Considering the results obtained using this tool, we can infer the confidence level of the studies included. However, it is important to note that studies with good results with AMSTAR 2 may mask critical weaknesses at specific points in the studies [22].

### 2.7. Analysis of the Degree of Overlap in Studies

**Figure 1:**
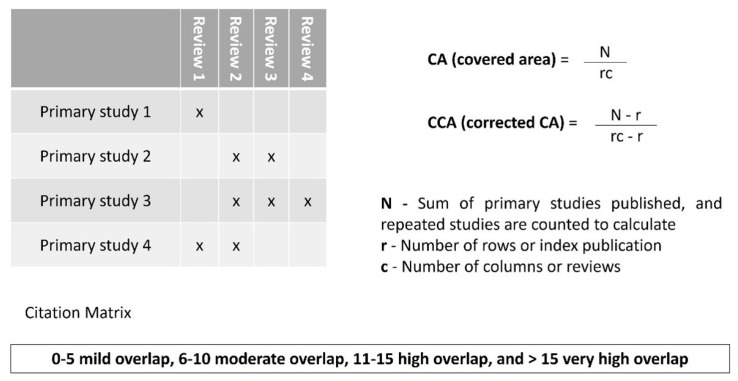
Citation matrix and calculation formulae.

To determine the overlap in studies across the systematic reviews, citation matrices were generated, and “corrected covered areas” (CCAs) were calculated (Figure 1). The overlap was considered as slight = 0–5; moderate = 6–10; high = 11–15; or very high > 15 [23,24,25,26].

## 3. Results

### 3.1. Study Selection

**Figure 2:**
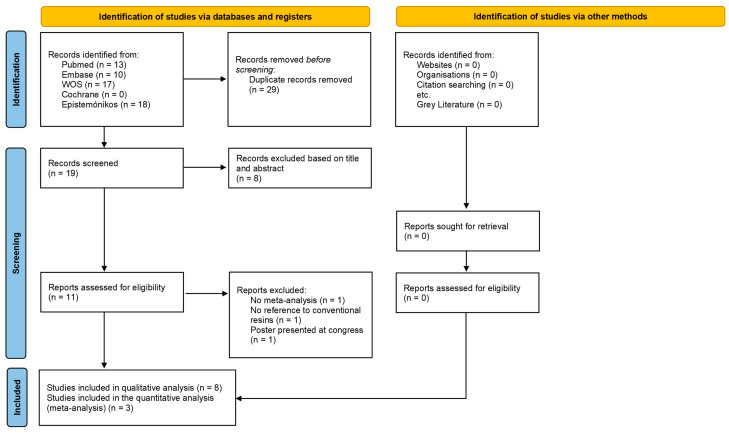
PRISMA flowchart.

The search of the different databases resulted in a total of 58 articles (Figure 2). The studies included were systematic reviews with meta-analyses demonstrating the comparison of the different characteristics of conventional resins and bulk-fill resins in posterior teeth restorations.

Of the 58 studies initially obtained in the database search, 29 studies were duplicated. Thus, the titles and abstracts of the 19 studies were reviewed in order to exclude studies that clearly did not fit the previously established inclusion criteria. This way, 11 studies were proposed for eligibility. These articles were read in full, and eight studies were included in the qualitative analysis of this review based on the inclusion and exclusion criteria (Table 3). Three articles were excluded (causes of exclusion can be found in Table 4).

### 3.2. Characteristics of the Included Reviews

The characteristics and results of the included reviews are shown in Table 5.

This umbrella review includes eight systematic reviews with meta-analyses [1,27,28,29,30,31,32,33]. By combining all the studies included in the different systematic reviews, it can be seen that this review is composed of 203 RCTs and 75 in vitro studies. Of these eight systematic reviews, only three are registered in PROSPERO [27,30,32]. Regarding the tools used for the analysis of bias in the studies, all eight articles used the Cochrane tool. The quality of the evidence obtained after the bias analysis of each study showed that four studies presented moderate quality [1,27,28,29] while another four studies presented low quality [30,31,32,33]. In all studies, the main objective was to compare conventional composite resins with bulk-fill composite resins regarding different properties.

Regarding the properties evaluated in the different studies, the total cumulative number of properties evaluated for all included studies was 39. The numbers of evaluated characteristics of the various studies were:

Arbildo-Vega et al. (2020)–11 characteristics [1]; Bellinaso, M. D. et al. (2019)–1 characteristic [27]; Cidreira Boaro et al. (2019)–9 characteristics [28]; Gerula-Szymańska et al. (2020)–1 characteristic [29]; Kruly et al. (2018)–5 characteristics [30]; Meereis et al. (2018)–1 characteristic [31]; Veloso et al. (2018)–8 characteristics [32]; and Zotti et al. (2021)–3 characteristics [33].

Among the various articles included in this review, there were common properties present. The evaluated properties that appeared most frequently in the included studies were the following: the appearance of secondary caries or caries recurrence appeared in four different studies [1,30,32,33]; marginal discoloration was appraised in four studies [1,30,32,33]; similarly, marginal adaptation was recorded in four different studies [1,30,32,33]; and postoperative sensitivity was measured in three different studies [1,30,32]. It should be noted that three of the studies only evaluated one single property [27,29,31].

As for the main results obtained in each of the studies, they demonstrated a trend of similarity between conventional resins and bulk-fill resins in terms of clinical performance.

Arbildo-Vega et al. (2020) [1] did not find significant differences between the two types of composite resin after evaluating the different characteristics related to clinical performance, regardless of the type of restoration (class I/II and non-carious cervical lesions), the type of dentition (primary or permanent) and the technique used (incremental, bulk or two-step bulk).

For Bellinaso et al. (2019) [27], when comparing the time required to perform a restoration between the two types of composite resin, demonstrated that the full-body bulk-fill composite resins required a shorter chair time to perform restorations in posterior teeth than conventional resins applied incrementally, thus confirming one of the main characteristics that make this type of composite resin desirable. However, flowable bulk-fill composites do not show the same evidence.

Cidreira Boaro et al. (2019) [28] concluded that bulk-fill resins perform similarly or better than conventional resins. However, although no differences were found between the two materials in terms of flexural strength and fracture strength, in terms of polymerisation stress and cusp deflection, they were lower when referring to bulk-fill resins. It should also be noted that, in this study, the volumetric shrinkage, degree of conversion and microhardness varied in their results according to the thickness and/or viscosity of the materials.

Gerula-Szymańska et al. (2020) [29] presented as the main result a similarity in marginal integrity between different types of bulk-fill resins (flowable and packable). It should be considered that the marginal integrity was analysed based on the restoration margin of two types of tissue: enamel and dentin.

Kruly et al. (2018) [30] did not demonstrate any statistically significant differences in any of the characteristics they assessed. In this systematic review, although there are studies that favour either bulk-fill resins or conventional resins in the different properties evaluated, in none of them is the difference sufficiently consistent to make a conclusion. Thus, this study concludes that bulk-fill resins present a similar clinical performance to restorations with conventional composite resins.

Meereis et al. (2018) [31] compared the polymerisation shrinkage stress in different materials, including bulk-fill resins. This study showed that bulk-fill resins demonstrate better potential for reduced shrinkage stress, especially if materials with a low modulus of elasticity are used, hence favouring the use of fluid materials instead of materials with higher viscosities. However, the authors also list other studies that contradict this statement. Thus, they conclude that bulk-fill resins showed only a moderate potential in reducing mechanical stress.

Veloso et al. (2018) [32] divided bulk-fill resins into two groups (base/flowable and full-body/sculptable), and these were independently evaluated against conventional composite resins. The parameters evaluated in this study were anatomic form, marginal discoloration, secondary caries, composite resin fracture, tooth fracture and postoperative sensitivity. The results showed no significant differences, either with the base/flowable bulk-fill or with the full-body/sculptable bulk-fill. This way, the clinical performance of bulk-fill composite resins is comparable to conventional resins in direct posterior restorations.

Finally, in Zotti et al. (2021) [33], bulk-fill resin restorations showed a 5.1% reduction in the risk of marginal discoloration and 1.4% of secondary caries while demonstrating a 6.5% increase in the risk of marginal misfit when compared with conventional composite resins. However, the author notes the possible low evidence of the meta-analysis performed and mentions that a possible risk of associated bias is present.

### 3.3. Meta-Analysis

**Figure 3:**
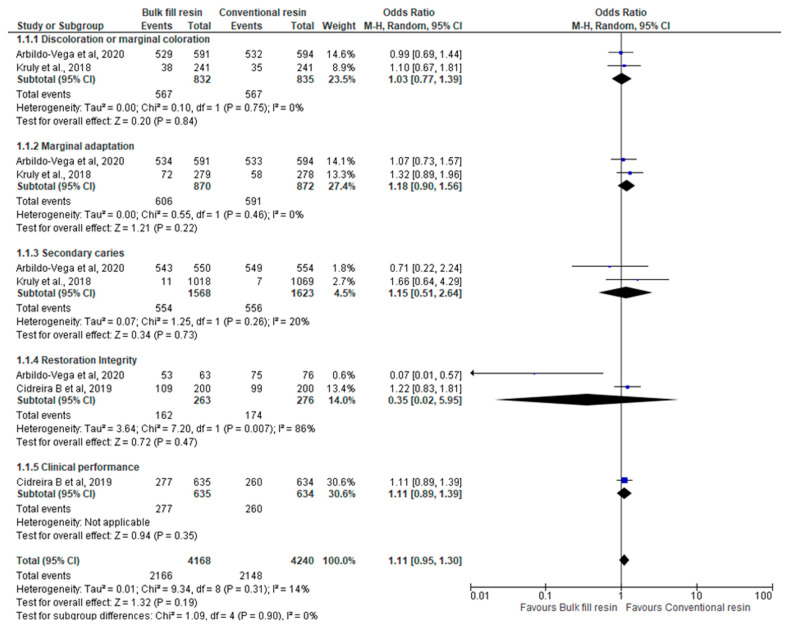
Outcomes measured by counting events (restoration loss/fail) [1,28,30].

In the quantitative analysis (meta-analysis), only three studies were included (Figure 3).

The exclusions in three studies were due to the lack of quantitative data comparing bulk-fill resins and conventional resins, namely in Gerula-Szymańska et al. (2020) [29], Meereis et al. (2018) [31] and Veloso et al. (2018) [32]. In the study by Zotti et al. (2021) [33], there was no report of the number of events in each group, only the total number of teeth included. In another study, by Bellinaso et al. (2019) [27], the final total mean and the respective standard deviation, essential data for the meta-analysis, were not identified since the results of this study were presented in mean difference.

In the evaluation of discoloration or marginal staining, the type of composite resin used seems to be negligible.

Regarding the marginal adaptation, the type of composite resin used seems to be irrelevant. However, both previous meta-analyses report a trend towards better results with conventional resin, although none of the results are statistically significant. The overall result is also not statistically significant, but it appears to be about four times more likely to obtain a favourable result with a conventional resin than with a bulk-fill resin.

Concerning the appearance of secondary caries, the results with conventional resin or bulk-fill resin are similar.

In assessing the restoration integrity, the type of composite resin used also appears to be irrelevant, with similar clinical results for both types of composite resin.

Regarding clinical performance, we only have information from one study, which does not demonstrate statistical significance.

Overall, although without statistical significance, the confidence interval for the OR (odds ratio) is most favourable to the use of conventional resin, as it is about five times more likely to obtain a good result with conventional resin than with bulk-fill resin.

### 3.4. Quality of the Included Reviews

The quality assessment of the selected studies is presented in Table 6.

All studies presented information related to the PICO question applied inclusion criteria and used comprehensive research. The study selection was performed in duplicate in all studies; however, data extraction was not performed in duplicate in two of the studies [28,31]. The list of excluded studies was not presented in five reviews [1,27,28,29,33]. The description of included studies was performed in all studies. All articles presented the assessment of the risk of bias of the included studies, as well as the results of the statistical combination, while none of the articles referred to the funding of the included studies. The effect of ROB on the statistical combination was not shown in two of the included articles [1,27] and ROB was not mentioned in the discussion of three of the articles [1,28,29]. Heterogeneity was discussed in all articles. Only one review did not present a publication bias analysis [27]. Finally, two of the studies did not mention any type of publication funding [27,31].

**Figure 4:**
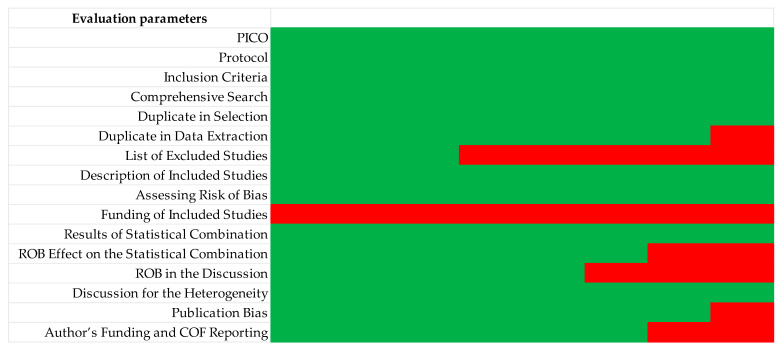
Overall quality. (Green—high quality; Red—low quality).

Therefore, with the application of the criteria of the AMSTAR 2 tool, one review was considered of very low quality [28], four reviews qualified as low quality [1,27,29,33] and three were considered of moderate quality (Figure 4) [30,31,32,33].

### 3.5. Degree of Umbrella Review’ Overlap

In eight systematic reviews selected for this umbrella, a total of 236 primary studies were included. According to the table in the supplementary information, in columns, eight systematic reviews were from recent to past years, and, in rows, 236 primary studies were ranked in the same way. According to the formulas, the CA and CCA were calculated as follows (Equations (1)–(3)) [23,24,25,26]:

**N = 278; r = 236; c = 8** (1)

**CA (covered area)** = N/rc = 278/236 × 8 = 278/1888 = **0.147** (2)

**CCA (corrected covered area)** = N – r/rc – r = 278 – 236/(236 × 8) – 236 = 42/ 1652 = **0.025** (3)

The results of CA and CCA were in the range between 0 and 5, which is considered a mild overlap. Thus, the low overlap of this umbrella review also confirms the need for conducting an overview such as this.

## 4. Discussion

The aim of this umbrella review is to synthesise the current literature on bulk-fill composite resins used in posterior tooth restorations, evaluate their performance and compare them with conventional composite resins based on systematic reviews with meta-analyses. All reviews present studies with very different follow-ups, which are from a minimum of 6 months to a maximum of 72 months, and most present results from 12 to 36 months of follow-up.

In summary, the results presented in this review point to a similarity between bulk-fill resins and conventional resins regarding the mechanical properties evaluated. Of the different studies included, six of them reached this conclusion after analysing and evaluating the data collected from the meta-analyses [1,28,29,30,31,32]. The characteristics that showed the most evidence regarding the similarity between the two types of composite resins were discoloration or marginal staining, marginal adaptation, secondary caries and restoration integrity. However, one of the studies favoured bulk-fill resins when evaluating clinical performance, the restoration time in this case [27], and another study did not draw concrete conclusions since there was no consensus/standardisation in data collection, which made a meta-analysis with good evidence impossible [33].

In the study by Arbildo-Vega et al. (2020) [1], all characteristics included, the absence of fractures, discoloration or marginal staining, marginal adaptation, postoperative sensitivity, secondary caries, colour stability and translucency, surface texture, anatomical shape, adequate tooth integrity, restoration integrity and proper occlusion were statistically evaluated concerning three subgroups: type of restoration, type of dentition and type of technique. The results showed that there were no significant differences between conventional resins and bulk-fill resins. In addition, in the study by Kruly et al. (2018) [30], all elements subjected to statistical analysis, namely marginal adaptation, marginal discoloration, secondary caries, retention and postoperative sensitivity, demonstrated that the overall effect was not statistically significant, concluding that clinical performance was similar between the experimental bulk-fill resin and conventional resin. Likewise, Veloso et al. (2018) [32] also did not show statistically significant differences regarding the characteristics evaluated: anatomical shape, marginal adaptation, marginal discoloration, colour combination, surface roughness, caries recurrence, fracture or retention, or postoperative sensitivity. They concluded that the clinical performance of bulk-fill composite resins was comparable to conventional resins in direct posterior restorations.

Finally, in statistical terms, the study by Zotti et al. (2021) [33] did not present a conclusion, since the meta-analysis was compromised, as already mentioned. However, bulk-fill resins are reported to present a faster clinical procedure, without compromising long-term clinical success.

Regarding Bellinaso et al. (2019) [27], it was the only study included that showed a statistically significant difference that favoured bulk-fill resins in terms of restoration time. The study ended up comparing flowable bulk-fill resins and full-body bulk-fill resins. Flowable bulk-fill resins need a surface layer of conventional resin, as they have a low amount of filler material and are therefore more susceptible to wear. On the other hand, full-body bulk-fill resins only need a single increment of composite resin, that is, they do not need a layer of conventional resin placed on the surface.

Despite their different applications and properties, they each have their advantages. While flowable bulk-fill resins can adapt better to cavity walls without creating gaps, full-body bulk-fill resins are faster to apply (in a single increment) and have better mechanical characteristics, namely concerning wear resistance and microhardness [43,44,45].

In the study by Cidreira Boaro et al. (2019) [28], in which nine characteristics were evaluated, it was inferred that conventional resins and bulk-fill resins present similar behaviour in terms of polymerisation stress, cusp deflection, marginal integrity, degree of conversion, flexural strength and fracture strength [46,47]. However, microhardness was one of the exceptions, since it can be reduced in bulk-fill resin restorations with a thickness of less than 2 mm. Regarding the volumetric shrinkage, since it depends on the inherent viscosity of the material used, it showed different results between the two types of bulk-fill resins when compared with conventional resins [46,47,48]. Full-body bulk-fill resins showed similar shrinkage compared with conventional resins, while flowable bulk-fill resins showed less shrinkage. This is due to the differences in the composition of the two types of bulk-fill resin. While the flowable bulk-fill resins, which belong to the bulk-fill resins initially used, present greater translucency and a smaller concentration and size of the filler particles, resulting in a more fluid composite resin, full-body bulk-fill resins have been developed and synthesised through the use of new types of monomers, filler particles and photo initiator systems, creating a composite resin with similar shrinkage to conventional resins [48,49,50,51,52]. Another characteristic related to volumetric shrinkage is polymerisation stress. In this study, lower stress was observed in all bulk-fill resins compared with conventional resins. This characteristic can be influenced by numerous factors, either by the composition of the composite resin or by the type of cavity that needs to be restored [28]. However, the authors conclude that materials with lower stiffness allow part of the shrinkage stress to be dissipated and, consequently, present a lower polymerisation shrinkage stress. As regards cusp deflection, it is also reduced when using bulk-fill resins compared with conventional resins. This fact is related to the polymerisation stress that is generated at the interface between the restoration and the tooth. Lower stress in this area corresponds to a lower displacement of the remaining tooth structure. Thus, a strong relationship between cusp deflection and polymerisation stress is demonstrated. The study by Meereis et al. (2018) [31] also evaluated the polymerisation shrinkage stress in different materials and restorative options, demonstrating that bulk-fill resins have moderate potential in reducing mechanical stress. The study addressed the differences between low- and high-viscosity resins and their influence on polymerisation stress. Low-viscosity composite resins demonstrated greater fluidity and, thus, relieved stress during the restorative procedure; however, the fact that they presented a low amount of filler could increase the stress exerted during polymerisation. The same happened with high-viscosity composite resins which, unlike low-viscosity composite resins (flowable bulk-fill), have a high amount of filler; this promotes an increase in the modulus of elasticity and consequently an increase in stress. This study ends up corroborating the study by Cidreira Boaro et al. (2019) [28]. It concluded that, since bulk-fill resins have a low modulus of elasticity, they demonstrate better performance in reducing polymerization stress, which favours choosing fluid materials over materials with a higher viscosity component.

Regarding marginal integrity, due to the lower stress, bulk-fill resins are expected to have better marginal integrity than conventional resins. However, the study by Cidreira Boaro et al. (2019) [28] reported that the marginal integrity was similar for both types of composite resin. Thus, the study suggests that other factors may influence this result, such as the adhesive system used, the viscosity of the material, the restorative technique and the clinician’s experience. In the review by Gerula-Szymańska et al. (2020) [29], flowable and packable bulk-fill resins were evaluated concerning marginal integrity, with results of similar behaviour in class II cavity restorations, as concluded in the study by Bellinaso et al. (2019) [27]. Francesco et al., in their systematic review, evaluated the clinical performance of bulk fill resins in cementum margins and concluded that it was not possible to establish differences between these resins and conventional ones in the integrity of cementum margins [13]

The degree of conversion, in addition to depending on the characteristics of the materials, such as the type of monomers, the charge and the photo initiator systems, is also influenced by factors related to the polymerisation of composite resins, such as the curing light, the radiation exposure and the type of light curing. In the study by Cidreira Boaro et al. (2019) [28], a greater degree of conversion of bulk-fill resins than conventional resins was demonstrated, with materials of normal viscosity with any thickness and fluid materials with a thickness greater than 2 mm. The characteristic that may have contributed most evidently to this result was the greater translucency that bulk-fill resins present.

Mechanical properties are also fundamental in comparing these composite resins and their performance. However, no difference was observed regarding mechanical properties between bulk-fill resins and conventional composite resins. Regarding microhardness, this is an exception, as mentioned above, since conventional regular and flowable resins with a thickness greater than 2 mm show greater microhardness compared with bulk-fill resins. This result is mainly explained by the lower concentration of filler materials found in bulk-fill resins, particularly in flowable resins and with thicknesses up to 2 mm, where the degree of conversion is similar for both types of resin. Finally, regarding flexural strength and fracture resistance, the study by Cidreira Boaro et al. (2019) [28] indicates that bulk-fill resins behave similarly to conventional resins since both have demonstrated an extremely low heterogeneity (0%), regardless of whether they are regular or flowable resins. Thus, this study ends up concluding that the interactions of all these factors did not exert a significant effect on the performance of the materials in up to 10 years of follow-up.

The fact that this umbrella review was prepared through a comprehensive consultation of systematic reviews published in the literature, consistently and objectively following different protocols, proves to be one of its main strengths. As previously mentioned, the AMSTAR 2 tool was used to assess the quality of the included studies. Considering the risk of bias measured by the AMSTAR 2 tool, the funding of the included studies, the list of excluded studies and the ROB in the discussion were identified as the items that most contributed to the decrease in the quality of the studies.

Funding for included studies is the only item that was not present in any of the studies selected in this review. For this reason, and because it is a critical factor, in future reviews it should be included by the researchers since its absence compromises the results.

Regarding the list of excluded studies, this was not present in half of the studies selected for the review [1,27,28,29]. Given that the listing of excluded studies and their reason for exclusion are important factors, its inclusion in future studies is critical. The fact that the studies did not present this list may compromise the transparency of the methodology used.

Finally, the ROB in the discussion was not present in three of the studies [1,28,29]. Studies must present a discussion of the ROB to summarise and justify the inclusion of studies and, consequently, discuss the risk of bias.

However, it is important to emphasise that all studies [1,27,28,29,30,31,32,33] adequately demonstrated comprehensive research, the inclusion criteria, and the review protocol, contributing to the increase in the review’s overall quality.

Although only systematic reviews were included, and these are associated with greater scientific evidence, it is of the utmost importance to underline that the studies included in these reviews also have associated risks of bias. However, studies that presented meta-analyses were exclusively included, since the absence of these may represent a high risk of bias, thus compromising the quality of the review.

Regarding the meta-analysis performed, it can be concluded that among the characteristics evaluated, such as discoloration, marginal adaptation, secondary caries, restoration integrity and clinical performance, the results seem to be similar, regardless of the type of composite resin used.

The absence of statistically significant clinical differences reported in this umbrella review between conventional resins and bulk-fill resins proves to be favourable in clinical practice, since clinicians may choose to use a composite resin that is easier to handle and requires less procedure time, which is the case with bulk-fill resins. However, the overall meta-analysis, although without statistical significance, favours mostly the use of conventional resin, as it is about five times more likely to obtain a favourable result than bulk-fill resin. This result may be due to the lack of individualisation of the results of the two types of bulk-fill resins and the multiple and non-standard clinical protocols for the application of bulk-fill resins, which make them more susceptible to clinical errors than conventional composite resins.

This review has some limitations, namely the fact that it only presents one review in the clinical performance assessment. Another aspect that was not achieved was the evaluation of other parameters recommended by the Academy of Dental Materials, such as resistance to fatigue, abrasion, friction, or the modulus of elasticity. It would still have been very interesting to compare the two main types of bulk-fill composite resins, but the non-separation of data in several reviews and even in the primary studies did not allow us to carry out this analysis.

Thus, in the future, the methodology should be a focus on materials studies. With the standardisation of the methodology, it would be easier to compare the various materials both in in vitro studies and in clinical studies. The comparison between the two types of bulk-fill resins and the conventional resin would be very important to compare clinical performances and help the dentist in his clinical decision.

## 5. Conclusions

The scientific evidence obtained in this umbrella review demonstrates that bulk-fill composite resin restorations have a performance similar to conventional composite resin restorations. The evaluation of other aspects, such as those related to the long-term success of these restorations, needs more studies and investigation to determine the further clinical benefits of using these composite resins. The quality of the evidence from the included studies was considered moderate, and the risk of bias was low. However, it is necessary to implement standardisation of studies regarding the characteristics under evaluation and data collection to improve scientific evidence and facilitate subsequent data analysis.

## Figures and Tables

**Table 1:** PICO Question.

	PICO
Population	Patients with direct composite resin restorations on permanent posterior teeth
Intervention	Posterior teeth restored with bulk-fill resins
Comparison	Posterior teeth restored with conventional resins
Outcome	Performance of bulk-fill resins (marginal coloration, marginal adaptation, secondary caries, restoration integrity and clinical performance)

**Table 2:** Search Strategy used.

	Search Strategy
PubMed/MEDLINE	“bulk fill” OR “bulk-fill” OR “bulkfill” OR “low shrinkage resin*” Filters: Systematic reviews, meta-analysis
Embase	(‘bulk fill’:ti,ab,kw OR bulkfill:ti,ab,kw OR ‘low shrinkage resin*’:ti,ab,kw) AND ([systematic review]/lim OR [meta-analysis]/lim)
WOS	(“bulk fill” OR “bulk-fill” OR “bulkfill” OR “low shrinkage resin*”) AND (“systematic review” OR “meta-analysis”)
Cochrane Library	“bulk fill” OR “bulk-fill” OR “bulkfill” OR “low shrinkage resin*” Filters: Systematic reviews, meta-analysis
Epistemonikos	(title:((title:(bulk fill) OR abstract:(bulk fill)) OR (title:(bulk-fill) OR abstract:(bulk-fill)) OR (title:(bulkfill) OR abstract:(bulkfill)) OR (title:(low shrinkage resin*) OR abstract:(low shrinkage resin*))) Filters: Systematic reviews, meta-analysis

**Table 3:** Included studies.

Author/Year	Journal	Title
Arbildo-Vega et al., 2020 [1]	Polymers	“Clinical Effectiveness of Bulk-Fill and Conventional Resin Composite Restorations: Systematic Review and Meta-Analysis”
Bellinaso, M. D. et al., 2019 [27]	Journal of Investigative and Clinical Dentistry	“Do bulk-fill resins decrease the restorative time in posterior teeth? A systematic review and meta-analysis of in vitro studies”
Cidreira Boaro et al., 2019 [28]	Dental Materials	“Clinical performance and chemical-physical properties of bulk fill composites resin—a systematic review and meta-analysis”
Gerula-Szymańska et al., 2020 [29]	Dental Materials	“Marginal integrity of flowable and packable bulk fill materials used for class II restorations—A systematic review and meta-analysis of in vitro studies”
Kruly et al., 2018 [30]	PLOS ONE	“Meta-analysis of the clinical behaviour of posterior direct resin restorations: Low polymerisation shrinkage resin in comparison to methacrylate composite resin”
Meereis et al., 2018 [31]	Journal of the Mechanical Behaviour of Biomedical Materials	“Polymerisation shrinkage stress of resin-based dental materials: A systematic review and meta-analyses of composition strategies”
Veloso et al., 2018 [32]	Clinical Oral Investigations	“Clinical performance of bulk-fill and conventional resin composite restorations in posterior teeth: a systematic review and meta-analysis”
Zotti et al., 2021 [33]	European Journal of Dentistry	“Microleakage of Direct Restorations—Comparison between Bulk-Fill and Traditional Composite Resins: Systematic Review and Meta-Analysis”

**Table 4:** Excluded studies.

Author/Year	Reason for Exclusion
Cavalheiro, C. P. et al. (2021) [34] Delgado, A. H. S. et al. (2021) [35] Lima, R. B. W. et al. (2018) [36] Lopes, L. C. P. et al. (2020) [37] Splieth, C. H. et al. (2020) [38] Schwendicke, F. et al. (2016) [39] Maran, B. M. et al. (2020) [40] Morais Sampaio, G. A. et al. (2021) [41]	Title and abstract
Reis, A. F. et al. (2017) [14]	No meta-analysis
Ajaj, R. et al. (2021) [18]	No reference to conventional resins
Dukic W. et al. (2017) [42]	Poster presented at congress

**Table 5:** Characteristics of the included reviews.

Author/Year	Design	Number of Studies & Design	ROB	ROB Tool	Sample Size	Evaluated Properties	Main Results
Arbildo-Vega et al., 2020 [1]	SR/MA	16 RCT	Moderate	Cochrane Handbook	1915	Absence of fractures, absence of discoloration or marginal staining, adequate marginal adaptation, absence of postoperative sensitivity, secondary caries, adequate colour stability and translucency, proper surface texture, proper anatomical form, adequate tooth integrity, adequate restoration integrity, and proper occlusion	“The clinical performance of conventional resins and bulk resins for carious lesion restorations is similar.”
Bellinaso, M. D. et al., 2019 [27]	SR/MA	3 IV	Moderate	Cochrane tool	NR	Restorative time	“The use of a full-body bulk-fill resin composite requires a shorter time to perform restorations in posterior teeth than conventional resins placed incrementally. There is not enough evidence to draw the same conclusion regarding flowable bulk-fill resin composites.”
Cidreira Boaro et al., 2019 [28]	SR/MA	148 RCT	Moderate	Cochrane guidelines	NR	Clinical performance, volumetric shrinkage, polymerisation stress, cusp deflection, marginal integrity, degree of conversion, microhardness, flexural strength and fracture strength	“Laboratory studies show similar or better performance of bulk-fill materials compared to the traditional composite resins.(…) The only exceptions are the lower microhardness of bulk-fill materials with a thickness of less than 2 mm.”
Gerula-Szymańska et al., 2020 [29]	SR/MA	10 IV	Moderate	Cochrane guidelines	106	Marginal integrity	“The present review indicates that flowable and packable bulk fill composites present similar marginal integrity when used for the restoration of class II cavities.”
Kruly et al., 2018 [30]	SR/MA	21 RCT	Low	Cochrane Handbook	NR	Marginal integrity/adaptation, marginal discoloration, recurrent caries, retention of resin restorations, and post-operative sensitivity	“Bulk-fill composites present similar clinical performance when compared to conventional resin composites.”
Meereis et al., 2018 [31]	SR/MA	62 IV	Low	Cochrane guidelines	NR	Polymerisation shrinkage stress	“Low-shrink and bulk-fill materials (…) showed only moderate potential in reducing stress.”
Veloso et al., 2018 [32]	SR/MA	10 RCT	Low	Cochrane tool	941	Anatomical shape, marginal adaptation and discoloration, surface roughness, colour, secondary caries, loss of retention, fracture, and postoperative sensitivity	“The clinical performance of bulk-fill resin composites is comparable to conventional resins in direct posterior restorations.”
Zotti et al., 2021 [33]	SR/MA	8 RCT	Low	Cochrane guidelines	778	Marginal discoloration, marginal adaptation, and secondary caries	“Bulk-fill composites as reliable and effective materials for direct restorations. Moreover, their properties allow to speed up the chair-side process without undermining clinical success overtime.”

SR/MA—Systematic review/meta-analysis; RCT—randomised controlled trials; IV—in vitro; ROB—risk of bias; NR–Not Reported.

**Table 6:** Quality evaluation of the included reviews (AMSTAR 2).

Author/Year	PICO	Protocol	Inclusion Criteria	Comprehensive Search	Duplicate in Selection	Duplicate in Data Extraction	List of Excluded Studies	Description of Included Studies	Assessing Risk of Bias	Funding of Included Studies	Results of Statistical Combination	ROB Effect on the Statistical Combination	ROB in the Discussion	Discussion for the Heterogeneity	Publication Bias	Author’s Funding and COF Reporting	Overall Quality
Arbildo-Vega, 2020 [1]	Yes	Yes	Yes	Yes	Yes	Yes	No	Yes	Yes	No	Yes	No	No	Yes	Yes	Yes	Low
Bellinaso, 2019 [27]	Yes	Yes	Yes	Yes	Yes	Yes	No	Yes	Yes	No	Yes	No	Yes	Yes	No	No	Low
Cidreira, 2019 [28]	Yes	Yes	Yes	Yes	Yes	No	No	Yes	Yes	No	Yes	Yes	No	Yes	Yes	Yes	Very Low
Gerula-Szymańska, 2020 [29]	Yes	Yes	Yes	Yes	Yes	Yes	No	Yes	Yes	No	Yes	Yes	No	Yes	Yes	Yes	Low
Kruly, 2018 [30]	Yes	Yes	Yes	Yes	Yes	Yes	Yes	Yes	Yes	No	Yes	Yes	Yes	Yes	Yes	Yes	Moderate
Meereis, 2018 [31]	Yes	Yes	Yes	Yes	Yes	No	Yes	Yes	Yes	No	Yes	Yes	Yes	Yes	Yes	No	Moderate
Veloso, 2018 [32]	Yes	Yes	Yes	Yes	Yes	Yes	Yes	Yes	Yes	No	Yes	Yes	Yes	Yes	Yes	Yes	Moderate
Zotti et al., 2021 [33]	Yes	Yes	Yes	Yes	Yes	Yes	No	Yes	Yes	No	Yes	Yes	Yes	Yes	Yes	Yes	Low

COI—conflict of interest; ROB—risk of bias.

## Data Availability

https://estudogeral.uc.pt/handle/10316/104503.

## Supplementary Materials

The following supporting information can be downloaded at: https://www.mdpi.com/article/10.3390/polym15122613/s1, Table of Citation matrix.
