# Supplementary material for: Bulk-Fill Resins versus Conventional Resins: An Umbrella Review
Source: Polymers (Basel). 2023 Jun 8;15(12):2613. doi: 10.3390/polym15122613 (PMC10301558; doi:10.3390/polym15122613)
Supplement: Supplementary file 1 [file polymers-15-02613-s001.zip › polymers-2423728-supplementary.pdf]

Supplementary table– Citation Matrix

Numbe	Columns	1	2	3	4	5	6	7	8
Rows	Review	Arbildo-Vega et al., 2020 [1]	Bellinaso, M. D. et al., 2019 [27]	Cidreira Boaro et al., 2019 [28]	Gerula-Szymańska et al., 2020 [29]	Kruly et al., 2018 [30]	Meereis et al., 2018 [31]	Veloso et al., 2018 [32]	Zotti et al., 2021 [33]
	Number of Primary Studies	16	3	148	10	21	62	10	8
1	Akman et al., 2020	*							
2	Balkaya et al. 2020	*							*
3	Correia et al, 2020	*							
4	Frascino et al, 2020	*							
5	Al-Sheikh, 2019	*							
6	Alquadalhi, 2019			*					
7	Balkaya et al, 2019			*					*
8	Borges, 2019			*					
9	Canali et al, 2019	*		*					
10	Frascino et al, 2019			*					
11	Ewels, 2017			*					
12	Hayashi, 2019			*					
13	Lacerda, 2019			*					
14	Mergulhão, 2019			*					
15	Nascimento, 2019			*					
16	Par, 2019			*					
17	Rizzante, 2019			*					
18	Scribame, 2019			*					
19	Taubock, 2019			*					
20	Abassi, 2018			*					
21	Almeida-Junior, 2017			*					
22	Alsagob, 2018			*					
23	Arora, 2018			*					
24	Behery, 2018			*					
25	Cerda-Rtzo, 2018			*					
26	Cuevas-Suá, 2018			*					
27	Eisharkasi, 2018			*					
28	Fronza, 2018			*					
29	Gamarra, 2018				*				
30	Gonçalves, 2018			*					
31	Haak, 2018			*					
32	Heck et al, 2018	*		*					*
33	Jiekhet, 2018			*					
34	Kamalac, 2018			*					
35	Kessier, 2018			*					
36	Lee, 2018			*					
37	Lempel, 2018			*					
38	Meenakumari, 2018			*					
39	Nascimento, 2018			*					
40	Nowak, 2018			*					
41	Ólafsson, 2018			*					
42	Oliveira, 2018			*					
43	Oter et al, 2018	*		*					

44	Peutzfeldt, 2018			*					
45	Prager, 2018			*					
46	Politi, 2018			*					
47	Shafie, 2018			*					
48	Tekce, 2018			*					
49	Tsujimoto, 2018			*					
50	Yarmohamadi, 2018			*					
51	Warangkulkasemkit, 2018			*					
52	Yap, 2018			*					
53	Agarwai, 2017			*					
54	AlShaffi, 2017			*					
55	Algamalah, 2017			*					
56	Almeida-Junior, 2017			*					
57	Atabek et al, 2017	*		*				*	
58	Bayraktar et al, 2017	*		*				*	
59	Colak et al, 2017	*		*				*	*
60	Fronza, 2017			*					
61	Han, 2017			*					
62	Hegde, 2017			*					
63	Jung, 2017			*					
64	Karaman et al, 2017			*				*	
65	Kubo, 2017			*					
66	McHugh, 2017			*					
67	Miletic, 2017			*					
68	Moharam, 2017			*					
69	Mosharrafian et al, 2017		*	*					
70	Patnana, 2017			*					
71	Rodrigues, 2017			*					
72	Shibasaki, 2017			*					
73	Shahidi et al, 2017				*				
74	Silame, 2017			*					
75	Souza-Lima, 2017			*					
76	Taha, 2017			*					
78	Tekce, 2017			*					
79	Van Dijken et al, 2017	*		*				*	
80	Vianna-de-Pinho et al, 2017		*	*					
81	Yazici et al, 2017	*		*				*	*
82	Yu, 2017			*					
83	Al-Harbi et al, 2016			*	*				
84	Al Sunbul et al, 2016			*			*		
85	Alkurdi and Abboud, 2016							*	*
86	Atalay, 2016			*					
87	Bacchi et al, 2016						*		
88	Bartsch, 2016			*					
89	Bayraktar, 2016			*		*			
90	Behry, 2016			*					
91	Bijelic-Donovan, 2016			*					
92	De Assis et al, 2016			*	*				
93	El Gewazi, 2016			*					
94	Engelhardt, 2016			*					
95	Farahat, 2016			*					
96	Fernandez, 2016			*					
97	Gasparello, 2016					*			
98	Guo et al, 2016			*			*		

99	Han et al, 2016			*			*		
100	Isuffi, 2016			*					
101	Jawaed, 2016			*					
102	Karaman, 2016			*		*			
103	Kapoor, 2016			*					
104	Kelic, 2016			*					
105	Kim et al, 2016			*			*		
106	Lempel, 2016			*					
107	Nguyen, 2016			*					
108	Ozel, 2016			*					
109	Öztürk-Bozkurt, 2016			*					
110	Sampaio, 2016			*					
111	Schliebe, 2016			*					
112	Scotti, 2016			*					
113	Son, 2016			*					
114	Tsujimoto, 2016			*					
115	Van Dijken et al, 2016	*		*				*	*
116	Vinagre, 2016			*					
117	Yap, 2016			*					
118	Yasa, 2016			*					
119	Agarwal et al, 2015				*				
120	Aishall, 2015			*					
121	Al-Harbi et al, 2015				*				
122	Bacchi et al, 2015						*		
123	Beigi Burujeny et al, 2015						*		
124	Benetti et al, 2015			*	*				
125	Caixeta, 2015			*					
126	Francis, 2015			*					
127	Fronza et al, 2015			*			*		
128	Gonçalves et al, 2015						*		
129	Heintze et al, 2015			*	*				
130	Hirata, 2015			*					
131	Ibarra, 2015			*					
132	Jang et al, 2015			*			*		
133	Kalmowicz, 2015			*					
134	Kemaloglu, 2015			*					
135	Kim et al, 2015			*			*		
136	Marigo, 2015			*					
137	Marovic, 2015			*					
138	Nagi, 2015			*					
139	Öznurhan, 2015			*					
140	Par, 2015			*					
141	Podgorski et al, 2015a						*		
142	Podgorski et al, 2015b						*		
143	Rengo, 2015			*					
144	Rosatto, 2015			*					
145	Schmidt, 2015					*			
146	Taubock, 2015			*					
147	Tomaszewska, 2015			*					
148	Toz 2015			*					
149	Van Dijken et al, 2015								*
150	Vidhawan, 2015			*					
151	Zorzin et al, 2015			*			*		
152	Aleixo et al, 2014						*		

153	Attia, 2014					*			
154	Bacchi et al, 2014						*		
155	Beck, 2014					*			
156	Bucuta, 2014			*					
157	Campos et al, 2014			*	*				
158	Cantekin, 2014			*					
159	Do, 2014			*					
160	El-Dammanhoury and Platt, 2014			*			*		
161	El-Eraky, 2014					*			
162	Garcia, 2014			*					
163	Goracci, 2014			*					
164	Guler, 2014			*					
165	Koyuturk, 2014			*					
166	Leprince, 2014			*					
167	Mahmoud, 2014					*			
168	Müller, Karaman, 2014		*						
169	Palin et al, 2014						*		
170	Santos, 2014					*			
171	Scotti, 2014			*					
172	Taubock et al, 2014			*			*		
173	Van Dijken, 2014					*			
174	Walter, 2014					*			
175	Watts and Alnazzawi, 2014						*		
176	Yazici, 2014					*			
177	Arslan, 2013			*					
178	Aishall, 2013			*					
179	Baracco, 2013					*			
180	Efes, 2013					*			
181	Garaoush, 2013			*					
182	Gonçalves, 2013					*			
183	Szaloki et al, 2013					*			
184	Yamasaki et al, 2013						*		
185	Baracco, 2012					*			
186	El-Shafty, 2012			*					
187	Gao et al, 2012						*		
188	Gonçalves et al, 2012						*		
189	Leung and Bowman, 2012						*		
190	Li et al, 2012						*		
191	Liu et al, 2012						*		
192	Moorthy, 2012			*					
193	Oliveira et al, 2012						*		
194	Park et al, 2012a						*		
195	Park et al, 2012b						*		
196	Park et al, 2012c						*		
197	Satterthwaite et al, 2012						*		
198	Ye et al, 2012						*		
199	Boulden et al, 2011						*		
200	Schreck et al, 2011						*		
201	Gonçalves et al, 2011						*		
202	Ilie and Hickel, 2011						*		
203	Moraes et al, 2011						*		
204	Roggendorf et al, 2011				*				
205	Schmidt, 2011					*			
206	Xiong et al, 2011						*		

Supplementary table– Citation Matrix

207	Ye et al, 2011						*		
208	Arhun et al, 2010	*						*	
209	Boaro et al, 2010						*		
210	Cramer et al, 2010a						*		
211	Cramer et al, 2010b						*		
212	Gonçalves et al, 2010a						*		
213	Gonçalves et al, 2010b						*		
214	Manhart et al, 2010	*						*	
215	Marchesi et al, 2010						*		
216	Min et al, 2010						*		
217	Bottenberg, 2009					*			
218	Pfeifer et al, 2009						*		
219	Van Dijken, 2009					*			
220	Cadenaro et al, 2008						*		
221	Chappelow et al, 2008						*		
222	Garoushi et al, 2008						*		
223	Bottenberg, 2007					*			
224	Carioscia et al, 2007						*		
225	Charton et al, 2007						*		
226	Eick et al, 2007						*		
227	Efes, 2006					*			
228	Carioscia et al, 2005						*		
229	Lu et al, 2005						*		
230	Wilder et al, 2005						*		
231	Ernst et al, 2004						*		
232	Lu et al, 2004						*		
233	Ferracane et al, 2003						*		
234	Braga and Ferracane, 2002						*		
235	Condon and Ferracane, 2002						*		
236	Condon and Ferracane, 1998						*		
